# Enhancing backcross programs through increased recombination

**DOI:** 10.1186/s12711-021-00619-0

**Published:** 2021-03-09

**Authors:** Elise Tourrette, Matthieu Falque, Olivier C. Martin

**Affiliations:** 1 Université Paris-Saclay, INRAE, CNRS, AgroParisTech, GQE - Le Moulon, 91190 Gif-sur-Yvette, France; 2 Université Paris-Saclay, INRAE, CNRS, Univ. Evry, Institute of Plant Sciences Paris-Saclay (IPS2), 91405 Orsay, France; 3 Université de Paris, CNRS, INRAE, Institute of Plant Sciences Paris-Saclay (IPS2), 91405 Orsay, France

## Abstract

**Background:**

Introgression of a quantitative trait locus (QTL) by successive backcrosses is used to improve elite lines (recurrent parent) by introducing alleles from exotic material (donor parent). In the absence of selection, the proportion of the donor genome decreases by half at each generation. However, since selection is for the donor allele at the QTL, elimination of the donor genome around that QTL will be much slower than in the rest of the genome (i.e. linkage drag). Using markers to monitor the genome around the QTL and in the genetic background can accelerate the return to the recurrent parent genome. Successful introgression of a locus depends partly on the occurrence of crossovers at favorable positions. However, the number of crossovers per generation is limited and their distribution along the genome is heterogeneous. Recently, techniques have been developed to modify these two recombination parameters.

**Results:**

In this paper, we assess, by simulations in the context of *Brassicaceae*, the effect of increased recombination on the efficiency of introgression programs by studying the decrease in linkage drag and the recovery of the recurrent genome. The simulated selection schemes begin by two generations of foreground selection and continue with one or more generations of background selection. Our results show that, when the QTL is in a region that initially lacked crossovers, an increase in recombination rate can decrease linkage drag by nearly ten-fold after the foreground selection and improves the return to the recurrent parent. However, if the QTL is in a region that is already rich in crossovers, an increase in recombination rate is detrimental.

**Conclusions:**

Depending on the recombination rate in the region targeted for introgression, increasing it can be beneficial or detrimental. Thus, the simulations analysed in this paper help us understand how an increase in recombination rate can be beneficial. They also highlight the best methods that can be used to increase recombination rate, depending on the situation.

**Supplementary Information:**

The online version contains supplementary material available at 10.1186/s12711-021-00619-0.

## Background

Breeding schemes that are based on recurrent backcrossing result in the introgression of an allele from a donor parent at a target locus into the genetic background of a recurrent parent. Beyond the need to maintain the donor allele at the target locus in the progenies, such schemes have two aims: (1) reduction of the size of the segment from the donor parent around the target locus; and (2) recovery of the recurrent parent genomic background. These objectives are achieved by having multiple generations of backcrosses between the offspring of the previous cross and the recurrent parent [1]. Backcrossing has multiple uses in genetics and breeding, which range from validating a putative allelic effect to genetically improving agriculturally important species. Backcrossing aims at transferring a favorable allele of one or multiple loci, which were previously in a poor genetic background, into a better background. Examples include introgression of a resistance gene from a non-elite genotype such as a landrace, introgression of a transgene into a reference line (if there are multiple loci, the process is called gene pyramiding [2]), unraveling the genetic architecture of a quantitative trait, testing for additivity of the effects of quantitative trait loci (QTL), or increasing the precision in QTL mapping [1, 3]. In the absence of any selection, the expected proportion of the donor genome will be halved at each generation. However, since the target locus is linked to its neighboring loci, the selection at the target locus will generally also select for the donor genome around this locus. As a result, the proportion of the donor genome will decrease less for the chromosome that carries the target locus (carrier chromosome) than for the others. This is the so-called linkage drag problem [4–6]. It is possible to accelerate the return to the recurrent genome and to reduce the linkage drag by exploiting markers both in the genetic background and close to the target locus [7–10]. Reduction of the linkage drag around the target locus depends on recombination rate, i.e., in the absence of recombination events, linkage drag will extend to the whole carrier chromosome. Since there is almost always some recombination, one can use multiple generations to accumulate recombination events (that is true even in the presence of crossover interference which prevents close-by crossovers from occurring during the same meiosis; indeed, crossovers are independent if one considers different meioses and thus different generations). Hence, it is possible to obtain crossovers that flank closely the target locus (on both sides) if a sufficient number of generations is used, essentially independently of the presence of crossover interference.

There are a number of methodological studies on backcross programs that have investigated the role of population size or location of markers. For instance, several authors have considered how to optimize the positions of a limited number of markers that flank the target locus [8, 11]. In particular, [12] and [13] concluded that the larger the population, the closer the markers should be to the target locus. Frisch and Melchinger [14] theoretically studied the proportion of donor genome at one generation according to the genetic map and markers used. Rodolphe et al. [15] focused on the parental composition of the chromosomes, *i.e.*, they considered the statistics of the mosaic chromosome structure as a function of generations, and reported the distribution of the sizes of the chromosomal blocks coming from the donor or the recurrent parent by assuming two models of recombination: recombination without interference and recombination with complete interference, *i.e.,* one crossover per chromosome at each generation.

Recombination events that arise during a backcross program will influence the extent to which the return to the recurrent genome is possible. Thus, in the current work, we investigated whether an increase in recombination rate might speed up introgression schemes.

A few experimental techniques have been developed to increase recombination rates or modify recombination landscapes. One method knocks-out anti-crossover genes (such as in *Arabidopsis thaliana* [16] and in pea, rice and tomato [17]), and another one is based on modifying the ploidy levels in *Brassica rapa* [18]. Both of these methods lead to many-fold increases in crossover rates and, interestingly, the second one also affects the recombination profiles by adding crossovers to the crossover-poor regions. More modest increases (up to two-fold) have been obtained through over-expression of pro-crossover genes (in *A. thaliana* [19, 20]). A completely different approach consists in targeting crossovers at specific genomic locations by transgenesis [21], which increases recombination rates considerably but only in very small regions. Beyond these methods that manipulate recombination rate by biotechnological means, it is possible to exploit natural variations in recombination rate. These arise as differences between male and female meiosis (for instance as shown in *A. thaliana* [22] and in barley [23]), as differences in genetic background [24, 25] or genetic control [26, 27], or as responses to different environmental conditions [28, 29]. Blary and Jenczewski [30] provide a comprehensive review of the possibilities to modify recombination rate. In *Arabidopsis thaliana*, one of the double-mutants of anti-crossover genes increased the recombination rate 7.8-fold [16] via the production of additional non-interfering crossovers. This decrease in interference increases the probability that, in a single meiosis, two crossovers occur close to each other, and for our purposes potentially on both sides of the target locus, in backcross schemes. Such a property may speed up the reduction of the linkage drag since it could bypass the need to select for a crossover on each side of the target locus in different generations (*i.e.*, what is normally obtained in two generations could perhaps be obtained in one generation).

In the current work, we used an in silico approach to determine the impact of increasing recombination rate and modifying recombination landscapes in backcross programs, by focusing on the objective of recovering as much as possible of the recurrent genome. Specifically, we simulated backcross programs under normal and increased/modified recombination schemes following the experimentally-observed changes within the first two approaches. These schemes respectively use (1) mutants of anti-crossover genes, and (2) modifications of the ploidy level; hereafter, we shall refer to method (1) as HR for “HyperRecombinant” and method (2) as Boost for boosted recombination. As our main focus, we use a “standard” backcross program in *Brassica rapa* in which after the third generation of the backcross (BC3), there is one generation of selfing (program BC3S1), but we also consider alternative programs. Based on our investigation of how successful such backcross programs are according to population size or position of the target locus, we find that modifying the recombination rate is generally quite advantageous if it changes the region that contains the target locus from cold to warm (or even hot) with respect to recombination.

## Methods

Using modeling and simulations, we investigated the effects of increasing recombination rate and/or modifying the shapes of recombination landscapes on a program that introgresses the allele of a donor parent at a target locus into a recurrent parent by successive backcrosses. All the corresponding results were obtained in the context of *Brassica rapa* landscapes (modified or not). Our computer codes were written in the programming language R [31], both for producing individual-based simulations of forward-in-time backcross breeding schemes and for all associated analyses.

### Recombination landscapes and simulation of crossover formation

**Fig. 1:**
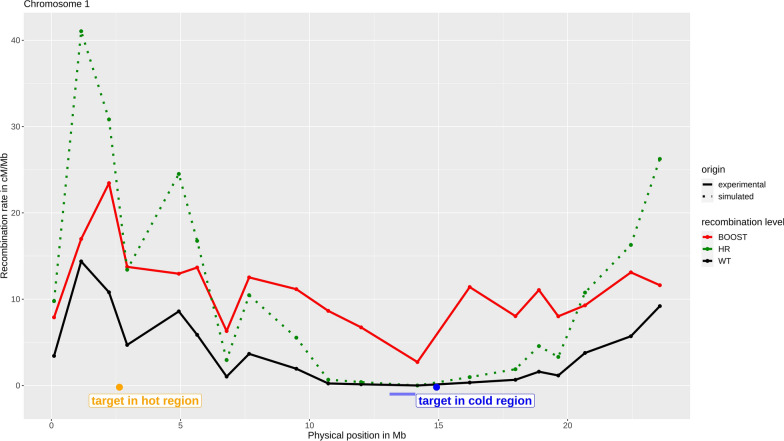
Recombination landscape of chromosome 1 of *Brassica rapa*. The WT recombination landscape is represented in black and the HR and Boost landscapes are in green and red, respectively. The solid lines represent the profiles obtained from experimental data (WT and Boost, data from [18]) and the dotted line is the simulated profile (HR) obtained by homothetic rescaling of the WT landscape. The yellow and blue dots represent the positions of the QTL in a hot and cold region, respectively (positions taken from [35]). The centromere position is represented by the blue bar [37]

Different scenarios of recombination in *Brassica rapa* were compared [for the associated recombination landscapes, see Fig. 1 and (see Additional file 1: Figure S1 and Additional file 2: Figure S2)]. The wild type (WT) scenario corresponds to the normal level of recombination whereas Boost and HR scenarios correspond to increased levels of recombination obtained by the two methods that are reviewed in the Background section and are presented below in detail in the context of *Brassica rapa*.

Under Boost, the modified recombination landscape has the key feature of producing a high increase in recombination rate (approximately 30-fold) in the pericentromeric regions, which are normally almost devoid of crossovers [18]. The associated landscapes were experimentally measured in the context of plants that have a non-standard karyotype, specifically with some chromosomes present as pairs (diploid state) and others present as singletons (haploid state). This mixed state is believed to perturb regulatory processes that otherwise maintain small numbers of crossovers. For our in silico study, we assumed that this modified landscape would arise in all the successive generations of the backcross program. In practice, this may mean that only a subset of the progeny (individuals having a sufficient number of chromosomes in the haploid state) can contribute to the program.

Under HR, we had to use proxies of the landscapes because they have not yet been measured in *Brassica rapa*. To do that, we considered the HR recombination landscapes in the species that have so far been studied as reported in [17]. We observed that HR enhanced recombination rate but did not change the *shapes* of the recombination profiles much, which corresponds rather well to a homothetic transformation multiplying all recombination rates by a constant. This qualitative trend is illustrated in Figure S3 [see Additional file 3: Figure S3] in the case of rice in which we compared the measured HR landscapes [17] to those obtained by homothetic transformations of the WT landscapes. As a result, the pericentromeric regions remain relatively poor in crossovers. These observations led us to take as proxies of the (unknown) HR landscapes, those that were obtained by multiplying the WT landscape by a constant that was different for each *B. rapa* chromosome. These constants were chosen such that the chromosome-wide increase in recombination rate was the same as that obtained with Boost; thus, the increase in recombination rate was set to $L_{{G_{Boost} }} /L_{{G_{WT} }}$, $L_{G}$ denoting the corresponding genetic lengths (see Table 1 and [Additional file 4: Table S1] for the genetic lengths under normal and increased recombination rates, for the female and male meiosis, respectively). As a consequence, by considering that Boost and HR reached the same global increase in recombination rate, we were able to observe the effects of changing or not the shape of the recombination profiles on recurrent backcrossing programs, when increasing the recombination rate.

**Table 1:** Recombination parameters for the *B. rapa* female recombination map

Chromosome	WT			Boost			HR		
	Genetic length	*p*	*nu*	Genetic length	*p*	*nu*	Genetic length	*p*	*nu*
A01	92.9	0.864	6.97	265.2	1	1.887	265.2	0	1
A02	78	0.97	6.139	260.7	1	1.479	260.7	0	1
A03	102.9	0.901	7.207	396.3	1	2.392	396.3	0	1
A04	56.2	0.93	18.576	206.5	1	1.946	206.5	0	1
A05	95.4	0.935	12.934	263.3	1	2.263	263.3	0	1
A06	105.7	0.936	8.814	300.9	1	3.084	300.9	0	1
A07	74.1	0.95	13.001	318.3	1	1.64	318.3	0	1
A08	56.8	0.929	12.185	225.8	1	2.036	225.8	0	1
A09	103.9	0.966	4.895	394.7	1	2.418	394.7	0	1
A10	55.8	0.919	63.778	143.1	1	2.362	143.1	0	1

Recombination parameters used for the *B. rapa* female recombination map. Parameters are: the genetic lengths, in centiMorgan, the proportion *p* of interfering crossovers and the shape parameter, *nu*, of the gamma distribution used to draw the inter-crossover distances under interference. These parameters are defined for a normal recombination rate (wild type, WT) and for increased recombination, either under Boost or HR. The values of *p* and *nu* are from [18] for WT and Boost but for HR we assumed no interference (*p* = 0, *nu* = 1).The genetic lengths under HR were set to the values measured under Boost

Since we had the physical positions of the target locus and of genome-wide distributed markers, it was necessary to calculate their genetic positions in each case (WT, Boost and HR). Using the different recombination landscapes, we interpolated the relationships between genetic and physical positions using splines (R function smooth.spline with spar = 0.1 to remove noise in the data). In our comparisons of these three cases, the genetic positions of the markers changed whereas their physical positions remained constant.

Meiotic recombination was simulated by generating crossovers in the genetic space. The recombination process was modeled using the two-pathway model [32–34], under which class I (interfering) and class II (non-interfering crossovers) are present at the same time. For the two classes of crossovers, the genetic distance between two adjacent crossovers of the same class is drawn from a gamma distribution, with shape parameter *nu*. In the case of non-interfering crossovers, *nu* = 1, corresponding to the model of Haldane under which the genetic distances are drawn from an exponential distribution. The proportion of crossovers that are interfering is given by the parameter *p*, and the expected total number of crossovers is given by the genetic length in Morgan.

The genome-wide markers were regularly spaced along the chromosomes (in physical coordinates), *i.e.*, one marker every 250 kb (4 per Mb) in the genome-wide background and 100 times more in a 2-Mb interval around the target locus (one every 2.5 kb) in order to be more precise during the foreground selection.

The target locus was located either in a cold region (at position 15 Mb, with a recombination rate of about 0.1 cM/Mb in the WT, 5.9 cM/Mb under Boost and 0.4 cM/Mb under HR), or in a hot region (at position 2 Mb, with a recombination rate of about 7.3 cM/Mb in the WT, 17.9 cM/Mb under Boost and 20.9 cM/Mb under HR) on chromosome 1 (these physical positions for the QTL are from [35], see Fig. 1).

### Backcross breeding schemes

**Fig. 2:**
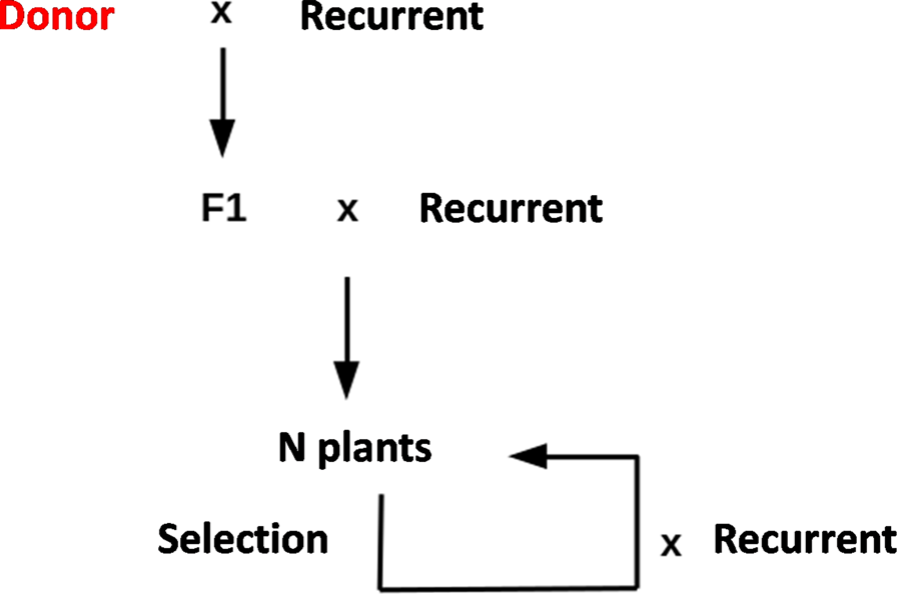
Scheme of a general introgression breeding program using successive backcrosses. The scheme displays the initialization and recurrent part of backcross programs, to which is generally added one generation of selfing referred to as BCnS1

In the first generation of the backcross schemes (Fig. 2), we crossed a donor parent that had the desired allele at the target locus to a recurrent parent that had the desired genetic background. Following this cross, which produces an F1, we simulated a succession of backcrosses between one (or more) offspring of the previous cross and the recurrent parent. Across successive generations, our aim was to recover as much as possible the recurrent background while keeping the donor allele at the target locus.

For the reference situation, which we present in detail in the main part of this paper, we used three or four generations of backcross followed by one generation of selfing. While the generations of backcrosses were obtained by using the female recombination maps (and thus used the recurrent parent as the male), for the generations involving selfing, one of the gametes was generated using the female maps and the other using the male maps. For better theoretical understanding, an additional situation of nine generations of backcross, followed by an additional generation of selfing, was also studied. At each generation, the population consisted of 400 plants (additional situations used 200 or 1000 plants). At each generation, one “best” plant was selected to be backcrossed to the recurrent parent at the next generation (we also considered the case of selecting the top 1% of the best plants).

To select the best individual, we used a two-step process. First, we pre-selected the plants that carry the donor allele at the target locus (thus about half of the population was kept in this step). For the second step, the selection criterion depended on the generation considered. In BC1 and BC2 (first and second generation of backcross after the initial cross between the donor and the recurrent), the plant with the closest crossover to the target locus was kept (in BC2, the side of the crossover was the opposite of that obtained in BC1) in order to reduce the linkage drag (foreground selection). In later generations, for each individual, we determined the proportion of donor genome, genome-wide, and the plant with the lowest proportion was kept. This reduction of the donor genome helps to recover the recurrent genome (background selection). To take into account the fact that the best plant may be unavailable (death, sterility), we also looked at the effect of keeping the top 1% of the plants (the four best plants) instead of the best one.

### Analyses

The success of a backcross program depends on two factors: the reduction of the linkage drag around the target locus and the recovery of the recurrent genome in the genetic background. Hence, in order to assess the effect of increasing recombination rate on a backcross scheme, we quantified both factors. The linkage drag was evaluated using the length, in Mb, of the donor segment around the target locus. The recovery of the recurrent genome, as for the background selection, was measured using the proportion of donor genome, genome-wide (specifically, the weighted proportion of markers carrying the donor allele, each marker being weighted according to the size of the interval it represents and the number—0, 1 or 2—of copies of the donor allele it includes). To further assess the speed of the return to the recurrent genome in the genetic background, we calculated the proportion of donor genome that was due to the linkage drag, *i.e.* that was due only to the donor segment around the target locus (weighted number of markers in the linkage drag segment divided by the total weighted number of markers as above).

To take the stochasticity of the recombination process into account and thus of the backcross selection program, 500 replicates were generated for each situation and we calculated the mean over the different replicates as well as the 95% confidence interval ($1.96\sigma$, $\sigma$ being the standard deviation). The confidence intervals are displayed in the figures via error bars. The errors *on the mean* are generally very small and thus are not displayed; consequently, we can use these measured means with a high level of confidence to compare the *average* behavior of the different scenarios and extract rankings.

## Results

### Effect of increased recombination rate on the linkage drag

**Fig. 3:**
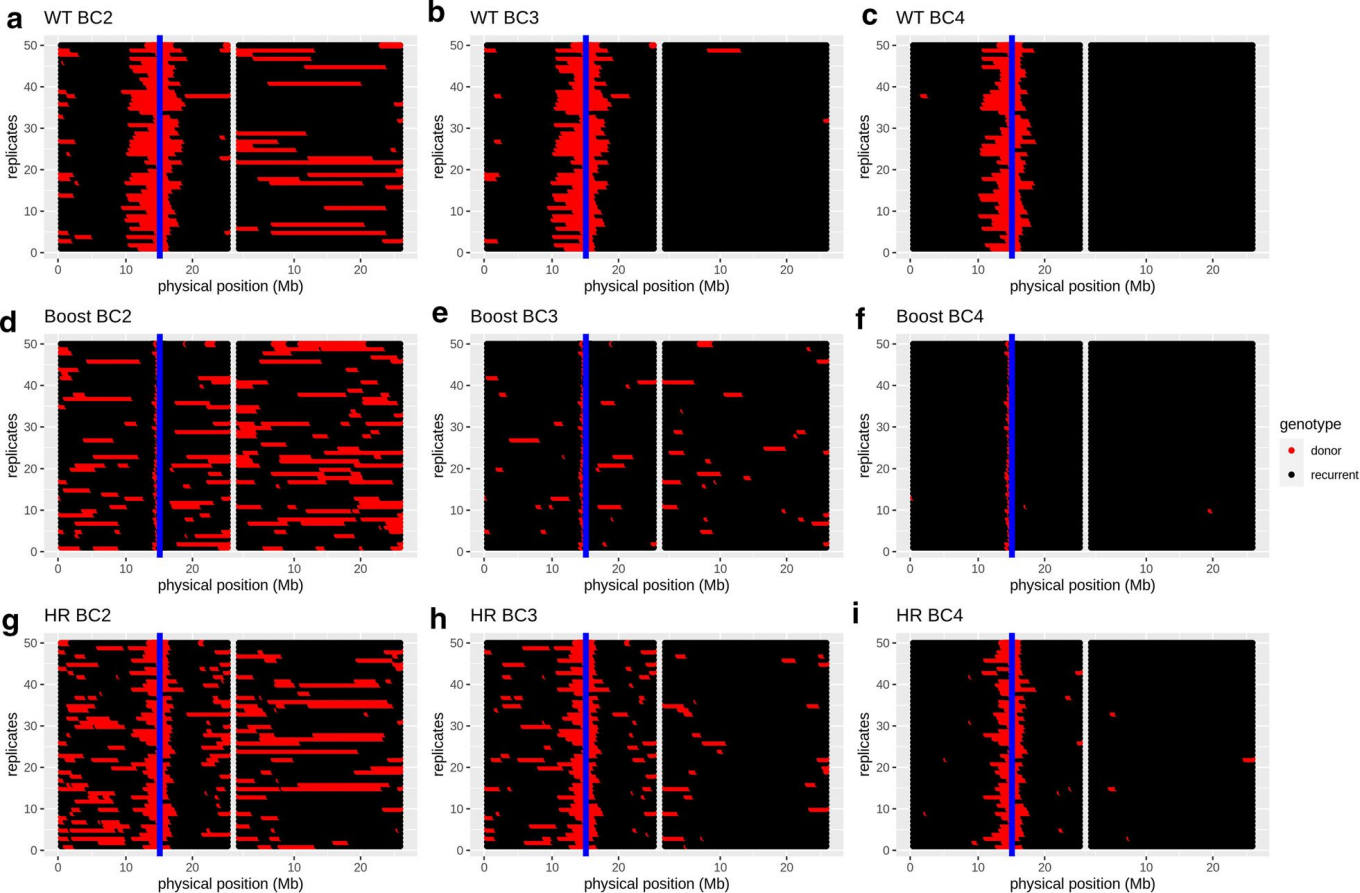
Graphical genotypes (genotypes at markers along chromosomes for 50 replicates of our simulations) for the best individual at generations BC2 (**a**, **d** and **g**), BC3 (**b**, **e** and **h**) and BC4 (**c**, **f** and **i**) under WT (top), Boost (middle) and HR (bottom) recombination, for chromosomes 1 and 2 of *B.rapa*. Heterozygous markers (donor/recurrent) are represented in red and homozygous markers for the recurrent allele are in black. The position of the target locus on chromosome 1 (left part of the graphical genotypes) is represented by a blue line, and chromosome 2 is represented on the right part of the graphical genotype, the two chromosomes being separated by a white line. Each of the individuals shown is that selected for its replicate i.e., the best one for that simulation

**Fig. 4:**
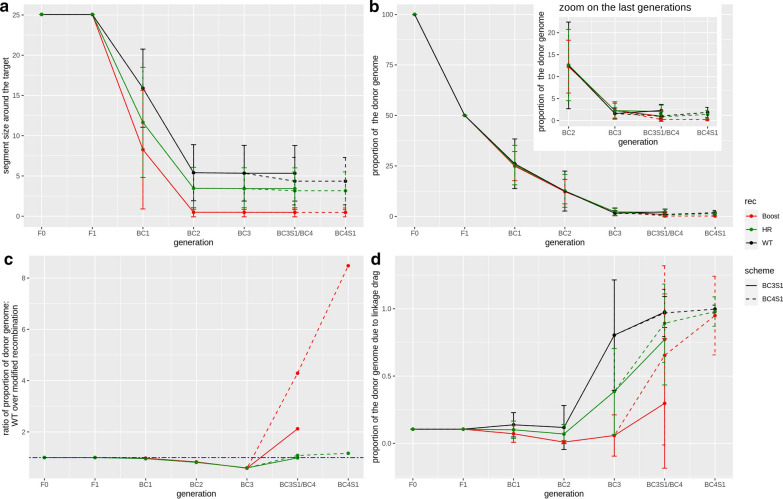
Effect of increased recombination rate for the introgression of a target locus in *B. rapa.* **a** Mean size of the heterozygous segment around the target locus in a cold recombinant region, in Mb, according to generations, in *B.rapa*. **b** Mean proportion of the donor genome, in percentage, according to generations. The insert represents a zoom on the last generations, from BC2 onwards. **c** Ratio of the mean proportion of the donor genome in the WT and of the corresponding proportion under Boost or HR according to generations. A value above 1 means that there is more remaining donor genome in the WT than under modified recombination (Boost or HR). **d** Mean proportion of the remaining donor genome that results from linkage drag, calculated as the part of the remaining donor genome that comes from the heterozygous segment around the target locus, according to generations. The measures in the WT are represented in black, those under Boost in red, and those under HR in green. Two selection schemes are shown: up to BC3S1, in solid lines, and up to BC4S1, in dashed lines. The generations BC3S1 and BC4 have the same position on the x axis. In the situations represented in this figure, the target locus is in a cold region, and there are 400 plants produced per generation. For the cross at each generation, the best individual is used, following the selection criterion appropriate for each generation (BC1 and BC2: foreground selection, and thereafter background selection). The error bars represent the confidence intervals at 95%

The linkage drag is monitored by the size, in Mb, of the donor segment around the target locus. Let us consider, first, the most interesting situation, *i.e.*, when the target locus is in a cold region. Figure 3 shows, via graphical genotypes, the realizations of typical segments whereas the corresponding mean sizes per generation are shown in Fig. 4a. In BC1 and BC2, the linkage drag decreases considerably because the selection focuses on its reduction by keeping the plants with the crossovers that are closest to the target locus. This decrease is much larger under Boost than under HR and is even larger when compared to the WT scenario: in BC1, the mean segment length is 15.9 Mb for WT, 11.6 Mb for HR and 8.3 Mb for Boost; and in BC2, the mean segment length is 5.4 Mb for WT, 3.5 Mb for HR and 0.49 Mb for Boost. For the later generations, since the selection focuses on the genome-wide recovery of the recurrent genome, the segment around the target locus hardly decreases with generations. For instance, in the BC3S1 program, the mean size of the donor segment at the target locus is 5.3 Mb in the WT, 3.4 Mb under HR, and 0.48 Mb under Boost. Adding one more generation (up to BC4S1) hardly affects these numbers, specifically, the mean size of the donor segment at the target locus is 4.3 Mb in the WT, 3.2 Mb under HR, and 0.47 Mb under Boost.

Overall, if very little recombination occurs around the target locus, that is detrimental for the selection program, although it includes 400 plants, *i.e.*, at the end of the BC2 generation, the donor segment around the target locus is about ten times larger in the WT scenario compared to Boost, which is due to the fact that, for Boost, recombination is significantly increased around the target locus (Fig. 1). In contrast, the advantage of HR compared to the WT scenario is definitely smaller, with a gain of only about 1.3. In the three scenarios overall, as population size increases, the linkage drag decreases (see Additional file 5: Figure S4a), *i.e.*, in BC3S1, with 200 individuals and 1000 individuals, the mean sizes of the donor segment at the target locus are respectively 6.9 and 3.5 Mb in the WT, 4.6 and 2.2 Mb under HR, and 0.81 and 0.23 Mb under Boost.

If more generations are added (see Additional file 6: Figure S5a), we observe a progressive decrease in the linkage drag. In BC9S1, although the selection from BC3 to BC9S1 is on the genome-wide proportion of the donor genome, it also acts on the donor segment that contains the target locus. For instance, in BC9S1, we obtain a mean size of the donor segment of 2.4 Mb in the WT, 1.7 Mb under HR and 0.14 Mb under Boost, and this decrease can be explained by the fact that most of the remaining donor genome is the result of linkage drag (*i.e.*, it is from the donor segment around the target locus, (see Additional file 6: Figure S5d), from BC5 onward, more than 90% of the donor genome is the result of linkage drag). Thus, selecting against the donor genome is similar to selecting for a decrease in linkage drag.

Let us consider now the situation of a target locus belonging to a hot region (see Additional file 7: Figure S6a), where recombination already occurs frequently in the WT. In that situation, the gain obtained from using Boost is much smaller: for the hot region chosen (Fig. 1), the mean segment size is 0.07 Mb for WT and 0.03 Mb for Boost in BC3S1, which corresponds to a reduction of linkage drag by a factor 2, *i.e.*, much lower than the factor 10 previously found when the target locus is in a cold region. In the case of HR, the segment length is 0.02 Mb under HR instead of 0.03 Mb under Boost in BC3S1 or 0.07 Mb under WT. Thus, enhanced recombination remains of interest to reduce the linkage drag but to a lesser degree than when the target locus is in a cold region.

### Effect of increased recombination on the recovery of the recurrent genome

#### For a reference situation

We followed the recovery of the recurrent genome via the (weighted) proportion of donor alleles remaining in the genome (see Methods). Figure 3 shows the graphical genotypes that provide insights on this issue and Fig. 4b shows the mean proportion at each generation. In the absence of selection on the proportion of donor genome (*i.e.,* no background selection), we expect the proportion of donor alleles to decrease by half at each generation, and this is what we observed up to BC2, *i.e.*, 100% in F0, 50% in F1, 25% in BC1 and 12.5% in BC2 for the three levels of recombination. However, once background selection is implemented (from BC3 onward), the proportion of donor genome decreased faster than in the absence of selection, *i.e.*, in BC3, 1.6% of the genome is from the donor in the WT, 2.2% under HR and 2.3% under Boost.

At the end of the selection program, the proportion of donor genome is lower under Boost than under HR and in the WT. Specifically, about twice as much donor genome remains in the WT and under HR than under Boost (Fig. 4c): when BC3S1 is reached, on average, 2.3% of the genome comes from the donor in the WT, 2.0% under HR but only 1.0% under Boost. It should be noted that the proportion of donor genome can *increase* after one round of selfing since some markers that were heterozygous will become homozygous for the donor allele. Overall, under Boost, the selection against the donor alleles is more effective than in the WT and under HR during the round of selfing, *i.e.*, while the proportion of donor genome increases in the WT and under HR, it decreases under Boost, which results in a lower proportion under Boost in BC3S1.

Let us consider now the situation where the target locus is in a region that is relatively hot for recombination in the WT (Fig. 1). In this case, we find that, in the WT, selection against the donor alleles is more effective than when recombination rate is increased (see Additional file 7: Figure S6b), whether under HR or Boost. Furthermore, under HR the proportion of donor genome is slightly lower than under Boost, e.g. in BC3S1, on average 0.06% of the genome comes from the donor in the WT, 0.45% under HR and 0.73% under Boost.

Although in the section “Effect of increased recombination on the linkage drag”, our results showed that increased recombination has benefits for decreasing linkage drag, it simultaneously hampers the recovery of the recurrent genome in the rest of the genome as shown in Figs. 3 and 4. This is true both for the chromosome that carries the target and for the other chromosomes. As illustrated in Figs. 3 and 4d, in the WT, most of the remaining donor genome is carried by the segment around the target locus (in BC3S1, 98% of the remaining donor genome is due to linkage drag in the WT and 77% under HR against 30% under Boost). Furthermore, Boost leads to many small segments of the donor genome being distributed across the different chromosomes. These segments are difficult to remove, which represents an intrinsic drawback of the increased recombination approach. Nevertheless, as we have seen before, adding one more generation of backcross cleans up the background considerably, in particular under Boost: for instance, in BC4S1 the percentage of the donor genome that results from linkage drag is then nearly 100% in the WT, 98% under HR and 95% under Boost.

#### General trends of the advantage of increased recombination

The first advantage of adding generations of backcross is that this is effective to eliminate even more of the donor genome. For instance, if the target locus is in a cold region, when the program goes up to BC4S1 the difference between the three levels of recombination becomes even more pronounced, with nearly ten times more donor genome remaining in the WT and under HR compared to Boost, *i.e.*, in BC4, 1% of the genome comes from the donor in the WT (1.8% in BC4S1) and 0.8% under HR (1.4% in BC4S1), while it represents 0.2% under Boost in BC4 and BC4S1. The advantage of Boost increases when adding more generations, but at a diminishing rate. For instance, the ratio between the mean proportion of donor genome in the WT and that under Boost is 4.3 in BC4, 9.4 in BC5, 11 in BC6, 12 in BC7, 13 in BC8 and 15 in BC9 (see Additional file 6: Figure S5c). The ratio of the mean proportion of donor genome under WT to that under HR is about 1 from BC4 to BC9, which is not surprising given that nearly all of the donor genome results from the linkage drag starting from BC4 in the WT and under HR, and starting from BC6 under Boost (see Additional file 6: Figure S5d).

Modification of other parameters in the backcross selection program led to similar trends. For instance, an increase in population size resulted in a better recovery of the recurrent genome (see Additional file 5: Figures S4b, c), *i.e.*, in BC3S1 for 200 individuals, on average 3.0% of the genome comes from the donor in the WT, 2.8% under HR and 1.3% under Boost, and for 1000 individuals, on average 1.5% of the genome comes from the donor in the WT, 1.3% under HR and 0.6% under Boost.

However, an increase in recombination rate does not always result in advantageous trends. For example, when the target locus is already in a relatively hot region in the WT, an increased recombination rate is disadvantageous (both under HR or Boost) since it increases the difficulty of cleaning up the genetic background, *i.e.*, in BC3S1, 12% of the remaining donor genome is in the background in the WT, 91% under Boost and 84% under HR.

### Selecting for the four best plants (top 1%) instead of the best one

All the previous results were obtained by assuming that the best individual is selected at each generation. Motivated by a form of bet-hedging, (the best individual could accidentally die or give rise to no seeds), we also looked at the effect of selecting the *four* best plants at each generation, *i.e.*, corresponding to the top 1% of individuals (see Additional file 8: Figure S7). In this case, the result is similar to that obtained when the best individual is selected, although this form of recurrent backcrossing is not as effective both in terms of reduction of the linkage drag and recovery of the recurrent genome. Specifically, when the four best plants are selected, in BC3S1, the average size of the donor segment around the target locus is 6.7 Mb in the WT, 4.9 Mb under HR and 1.6 Mb under Boost (compared to 5.3 Mb, 3.4 Mb and 0.5 Mb in the WT, and under HR and Boost, respectively, when the best individual is selected). Similarly, in these conditions, the average proportion of the donor genome is 2.9% in the WT, 2.7% under HR and 1.6% under Boost, compared to 2.3, 2.0 and 1.0% in the WT, under HR and under Boost, respectively, when the best individual is selected. We find that in the WT and under HR, the clean-up of the background is similar regardless of whether the best or four best plants are kept (at BC3S1, 98% in the WT and 77% under HR of the remaining donor genome is due to the donor segment around the target locus when the best plant is selected compared to 96% in the WT and 76% under HR when the four best plants are kept). In contrast, under Boost, selecting the four best individuals results in a lower relative contribution of the background: the donor segment around the target locus represents 30% of the remaining donor genome when selecting the best individual and 38% when the four best individuals are kept.

### Switching recombination landscapes between foreground and background selection

As previously observed, increasing recombination rate is particularly interesting to decrease the linkage drag (foreground selection) whereas a low recombination rate is rather efficient to clean-up the genetic background of any remaining donor genome. Thus, we examined the effect of an increase in recombination rate (Boost or HR) during the two generations of foreground selection followed by a switch back to normal recombination rate during background selection. Figure S8 (see Additional file 9: Figure S8) and Figure S9 (see Additional file 10: Figure S9) show the results when the target is in a cold or a hot region, respectively.

Regardless of whether the target locus is in a cold or hot region, switching back to the recombination rate at the WT level during background selection has almost no effect on linkage drag, *i.e.*, in BC3S1, when the target is in a cold region, the average donor segment size is 0.5 Mb under Boost and Boost-WT and 3.4 Mb under HR and 3.5 Mb under HR-WT. Similarly, when the target locus is in a hot region, the average donor segment size is 0.03 Mb under Boost and Boost-WT and 0.02 Mb under HR and HR-WT.

However, at the level of the global proportion of the donor genome, a small gain is observed when switching back to the level of recombination of the WT. When the target locus is in a cold region, in BC3S1, the proportion of the donor genome is 2.3% in the WT, 1.0% under Boost and 2.0% under HR, and is 0.7% under Boost-WT and 1.9% under HR-WT. In this situation, the Boost-WT strategy results in the highest overall performance. When the target locus is in a hot region, in BC3S1, the proportion of the donor genome is 0.06% in the WT, 0.7% under Boost and 0.5% under HR, and is 0.4% under Boost-WT and 0.3% under HR-WT. In this situation, the WT level of recombination gives the best results. Indeed, using enhanced recombination for BC1 and BC2 produces too many small donor segments genome-wide to compensate for the associated reduced size of the linkage drag, when compared to using simply the WT recombination landscapes for all generations.

## Discussion

The effect of increasing the level of recombination is strongest for regions that are initially poor in crossovers. Specifically, when the target locus is in a cold region (e.g. in pericentromeric regions), there is little recombination around the locus in the WT and under HR, whereas most of the region recombines under Boost, thus allowing for a sharp decrease in linkage drag during the foreground selection. In this situation, Boost is also globally beneficial, and even more when one more generation is added to the recurrent backcross program (using BC4S1 instead of BC3S1), which results in a fairly efficient removal of the donor genome during background selection.

In contrast, when the target locus is in a hot region, recombination at the WT level is sufficient to drastically decrease linkage drag during foreground selection. The situation in the WT is also better globally because, under increased recombination, many small donor blocks occur in the genetic background and this is disadvantageous during background selection [36]. Thus, when the target loci are in hot regions, increasing the level of recombination is generally detrimental. To be explicit, although it does decrease linkage drag (at the end of the foreground selection, in our simulations the mean donor segment size is 0.07 Mb in the WT, 0.03 under Boost and 0.02 under HR), it is not sufficient to compensate for the increased amount of donor genome in the genetic background.

By increasing the population size, it is possible for crossovers to occur closer to the target locus [12, 13], even in the WT and under HR, but never to the level reached when using Boost, in the range of population sizes studied here. However, let us note that under Boost, a change in population size has a smaller effect than in the WT and under HR, but overall, Boost remains the best.

We also found that it is possible to decrease linkage drag in the WT and under HR by adding more generations. At some point (around BC5), almost all of the remaining donor genome will correspond to the segment around the target locus. Thus, selection for a decreased proportion of donor genome will decrease the size of the donor segment around the target locus. This effect is particularly strong in the WT because the segment around the target locus is large and there is nearly no undesired donor genome in the genetic background. This property justifies the importance of foreground selection, *i.e.*, in a long-term backcross selection program, the amount of residual donor genome will be nearly completely determined by the quality of the foreground selection.

We also investigated whether switching the level of recombination during the backcross program could further improve the efficiency of the introgression. Indeed, we found that an increased level of recombination was beneficial during foreground selection to decrease linkage drag whereas maintaining a normal level of recombination was better for cleaning up the genetic background. When the target locus is in a cold region, switching from Boost to WT for the background selection indeed speeds up the decrease in donor genome (see Additional file 9: Figure S8). On the contrary, when the target locus is in a hot region, it is better to have the WT level of recombination throughout all generations rather than switching strategies between foreground and background selection (see Additional file 10: Figure S9).

Regarding potential impacts on real breeding programs, one relevant question is: can an increased level of recombination speed up backcross programs by reducing the number of generations used? Focusing on the main selection scheme of our work, namely foreground selection for two generations followed by one or two background selections and then by selfing, Fig. 4b and 4c show that, under Boost, BC3S1 is about twice as good as BC4S1 in the WT (and to a lesser extent, HR is also better than WT). Thus, under certain conditions, an increased level of recombination may speed up backcross programs while simultaneously improving performance. The advantage of an increased level of recombination is even more striking when considering the long backcross programs considered in Figure S5c (see Additional file 6: Figure S5c), which shows that Boost at BC4 outperforms all generations of WT including BC9S1. Other strategies may also exist for decreasing generation times thanks to increased recombination. For instance, foreground selection could be applied for a single generation, which would correspond to selecting double recombinants (crossovers on both sides of the target locus) in BC1. Clearly, the performance of such an approach will depend a lot on how the recombination landscape around the target locus is changed when going from WT to Boost but it may provide a practical way to speed-up backcross programs for introgressing segments that are in certain cold regions.

Although an increased level of recombination is likely to be advantageous in a number of situations, in practice application of these methods has some constraints. For Boost, an increased level of recombination is achieved by having some chromosomes in the triploid state, a characteristic that can be enforced through selection with the help, for instance, of flow cytometry measurements. Interestingly, since an increased level of recombination is mainly useful during foreground selection (BC1 and BC2), return to the WT level of recombination will occur spontaneously once triploid selection is removed. This advantageous property also holds for the HR approach that depends on having individuals that are homozygous for the HR allele (which is recessive). By using a non-HR recurrent parent after BC1, the recessive HR allele will first switch to the heterozygous state and then it will be eliminated, ensuring WT recombination landscapes for all the background selection generations.

## Conclusions

These simulations show that increasing the level of recombination for the introgression of a target locus leads to contrasted results: (1) for a target locus in a cold region, increasing the level of recombination decreases significantly the associated linkage drag while the successive generations of background selection adequately remove the rest of the donor genome; and (2) for a target locus in a hot region, increasing the level of recombination around the target locus is not necessary and an increase in the recombination level genome-wide will even be detrimental for eliminating the donor genome in other regions. Our findings help understand the conditions under which an increased level of recombination improves the efficiency of the introgression of a target locus by recurrent backcrossing, and also contribute to evaluate the potential advantages of the various techniques that may increase the level of recombination for crop breeding programs.

## Supplementary Information

**Additional file 1**:** Figure S1**. Female recombination landscapes for the 10 chromosomes of Brassica rapa. The WT, HR, and Boost recombination landscapes are represented in black, green and red, respectively. The solid lines represent the profiles obtained from experimental data (WT and Boost, data from [18]) and the dotted line is the simulated profile (HR). The centromere positions are represented by blue bars [37].

**Additional file 2**:** Figure S2**. Male recombination landscapes for the 10 chromosomes of Brassica rapa. The WT, HR and Boost recombination landscapes are represented in black, green and red, respectively. The solid lines represent the profiles obtained from experimental data (WT and Boost, data from [18]) and the dotted line is the simulated profile (HR). The centromere positions are represented using blue bars [37].

**Additional file 3**:** Figure S3**. Recombination landscapes for the 12 chromosomes of Oryza sativa. The WT and HR recombination landscapes are represented in black and green, respectively. Solid lines represent the landscapes obtained experimentally [17] and the dotted line the simulated. The centromere positions are represented by blue bars [38]. This figure shows the effect of HR recombination on the shape of the recombination landscape, as well as the relevance of a homothetic rescaling to approximate HR, thereby justifying this approach for simulating HR in B. rapa.

**Additional file 4**:** Table S1** Recombination parameters for the B. rapa male recombination map. Description: Parameters are: the genetic lengths, in centiMorgan, the proportion p of interfering crossovers and the shape parameter, nu, of the gamma distribution used to draw the crossovers under interference. These parameters are defined for a normal recombination rate (wild type, WT) and for increased recombination, either under Boost or HR. The values of p and nu are from [18] for WT and Boost whereas for HR, we assumed no interference (p = 0, nu = 1). The genetic lengths under HR were set to the values measured under Boost.

**Additional file 5**:** Figure S4**. Effect of the population size. (a) Mean size of the heterozygous segment around the target locus, in Mb, according to generations, in B.rapa. (b) Mean proportion of the donor genome, in percentage, according to generations. The insert represents a zoom on the last generations, from BC2 onwards. (c) Ratio of the mean proportion of donor genome under WT over the proportion under Boost or HR according to generations. A value above 1 means that there is more remaining donor genome in the WT than under modified recombination rates (Boost or HR). (d) Mean proportion of the remaining donor genome that results from the linkage drag, calculated as the part of the remaining donor genome that comes from the heterozygous segment around the target locus, according to generations. The measures for WT, Boost and HR are represented in black red and green, respectively. Different population sizes are shown: 200 plants per generation as dashed lines, 400 as solid lines, and 1000 as dotted lines. In the situations represented in this figure, the target locus is in a cold region, and the selection scheme goes up to BC3S1. The best individual is kept at each generation, following the selection criterion appropriate for each generation (BC1 and BC2: foreground selection, and thereafter background selection). The error bars represent the confidence intervals at 95%.

**Additional file 6**:** Figure S5**. Effect of the number of generations of backcross used. (a) Mean size of the heterozygous segment around the target locus, in Mb, according to generations, in B.rapa. (b) Mean proportion of the donor genome, in percentage, according to generations. The insert represents a zoom on the last generations, from BC2 onwards. (c) Ratio of the mean proportion of donor genome in the WT to the proportion under Boost or HR according to generations. A value above 1 means that there is more remaining donor genome in the WT than under modified recombination rates (Boost or HR). (d) Mean proportion of the remaining donor genome that results from the linkage drag, calculated as the part of the remaining donor genome that comes from the heterozygous segment around the target locus, according to generations. The measures for WT, Boost and HR are represented in black, red and green, respectively. The selection schemes go up to different numbers of generations: BC3S1 in solid lines, BC4S1 in dashed lines and BC9S1 in dotted lines. In the situations represented in this figure, the target locus is in a cold region and there are 400 plants per generation. The best individual is kept at each generation, following the selection criterion appropriate for each generation (BC1 and BC2: foreground selection, and thereafter background selection). The error bars represent the confidence intervals at 95%.

**Additional file 7**:** Figure S6**. Introgression in a hot vs cold region. (a) Mean size of the heterozygous segment around the target locus, in Mb, according to generations, in B. rapa. (b) Mean proportion of the donor genome, in percentage, according to generations. The insert represents a zoom on the last generations, from BC2 onwards. (c) Ratio of the mean proportion of donor genome in the WT to the proportion under Boost or HR, according to generations. A value above 1 means that there is more remaining donor genome in the WT than under modified recombination rates (Boost or HR). (d) Mean proportion of the remaining donor genome that results from the linkage drag, calculated as the part of the remaining donor genome that comes from the heterozygous segment around the target locus, according to generations. The measures for WT, Boost and HR are represented in black, red, and green, respectively. The target locus is either in a cold region (solid lines) or in a hot region (dashed lines). HR was not considered when the QTL was in a cold region since the effect of an increased recombination rate mainly influenced the linkage drag and the result under HR was not very different from that in the WT in the cold region. In the situations represented in this figure, there are 400 plants per generation and the selection scheme goes up to BC3S1. The best individual is kept at each generation, following the selection criterion appropriate for each generation (BC1 and BC2: foreground selection, and thereafter background selection). The error bars represent the confidence intervals at 95%.

**Additional file 8**:** Figure S7**: Effect of selecting the top 1% best plants rather than the best one. (a) Mean size of the heterozygous segment around the target locus, in Mb, according to generations, in B.rapa. (b) Mean proportion of the donor genome, in percentage, according to generations. The insert represents a zoom on the last generations, from BC2 onwards. (c) Ratio of the mean proportion of donor genome under WT to the proportion under Boost or HR according to generations. A value above 1 means that there is more remaining donor genome in the WT than under modified recombination rates (Boost or HR). (d) Mean proportion of the remaining donor genome that results from linkage drag, calculated as the part of the remaining donor genome that comes from the heterozygous segment around the target locus, according to generations. The measures for WT, Boost and HR are represented in black, red, and green, respectively. Either the best (solid lines) or the best four (top 1%; dashed lines) plants are kept at each generation. In the situations represented in this figure, the target locus is in a cold region, there are 400 plants per generations and the selection scheme goes up to BC3S1. The error bars represent the confidence intervals at 95%.

**Additional file 9**:** Figure S8**. Effect of switching recombination landscapes when the target locus is in a cold region (a) Mean size of the heterozygous segment around the target locus, in Mb, according to generations, in B.rapa. (b) Mean proportion of the donor genome, in percentage, according to generations. The insert represents a zoom on the last generations, from BC2 onwards. (c) Ratio of the mean proportion of donor genome in the WT to the proportion under Boost or HR according to generations. A value above 1 means that there is more remaining donor genome in the WT than under modified recombination rates (Boost or HR). (d) Mean proportion of the remaining donor genome that results from the linkage drag, calculated as the part of the remaining donor genome that comes from the heterozygous segment around the target locus, according to generations. The measures for WT, Boost and HR are represented in black, red, and green, respectively. The situations in which the recombination rate is switched between foreground (increased recombination) and background (normal recombination) selection are represented in purple for the switch Boost to WT, and in blue for the switch HR to WT. In the situations represented in this figure, the target locus is in a cold region, there are 400 plants per generations and the selection scheme goes up to BC3S1. The error bars represent the confidence intervals at 95%.

**Additional file 10**:** Figure S9**. Effect of switching recombination landscapes when the target locus is in a hot region. (a) Mean size of the heterozygous segment around the target locus, in Mb, according to generations, in B.rapa. (b) Mean proportion of the donor genome, in percentage, according to generations. The insert represents a zoom on the last generations, from BC2 onwards. (c) Ratio of the mean proportion of donor genome in the WT to the proportion under Boost or HR according to generations. A value above 1 means that there is more remaining donor genome in the WT than under modified recombination rates (Boost or HR). (d) Mean proportion of the remaining donor genome that results from the linkage drag, calculated as the part of the remaining donor genome that comes from the heterozygous segment around the target locus, according to generations. The measures for WT, Boost and HR are represented in black, red, and green, respectively. The situations in which the recombination rate is switched between foreground (increased recombination) and background (normal recombination) selection are represented in purple for the switch Boost to WT, and in blue for the switch HR to WT. In the situations represented in this figure, the target locus is in a hot region, there are 400 plants per generations and the selection scheme goes up to BC3S1. The error bars represent the confidence intervals at 95%.

## Data Availability

The parameters files for each simulation and the analysis scripts are included in the R package containing the simulation scripts. The scripts for the simulations can be downloaded as an R package on https://sourcesup.renater.fr/frs/download.php/latestfile/2217/CAREB_12.tar.gz.
