# Supplementary figures and images for: Enhancing backcross programs through increased recombination
Source: Genet Sel Evol. 2021 Mar 9;53:25. doi: 10.1186/s12711-021-00619-0 (PMC7941899; doi:10.1186/s12711-021-00619-0)

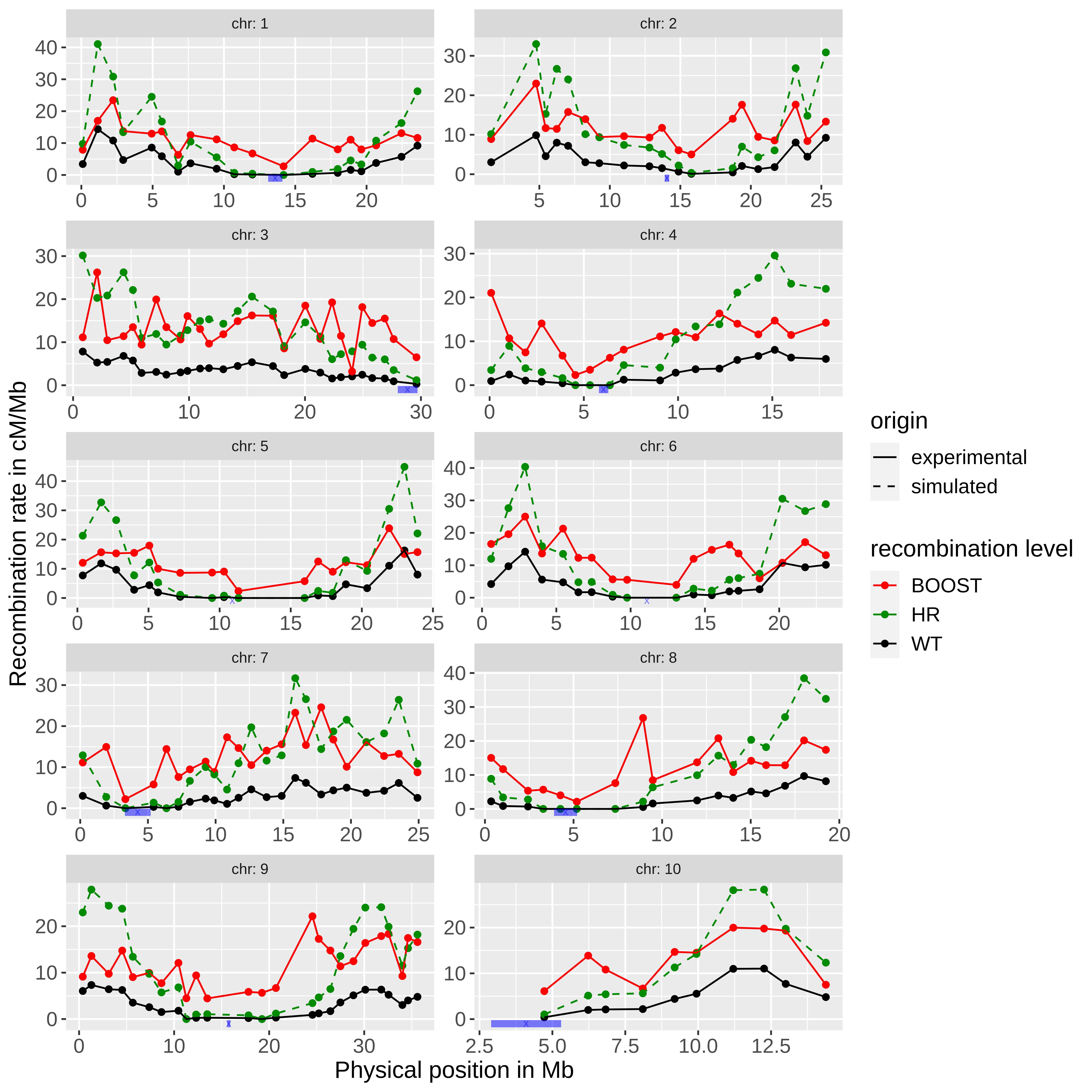

Supplement: Supplementary file 1 — Additional file 1: Figure S1. Female recombination landscapes for the 10 chromosomes of Brassica rapa. The WT, HR, and Boost recombination landscapes are represented in black, green and red, respectively. The solid lines represent the profiles obtained from experimental data (WT and Boost, data from [18]) and the dotted line is the simulated profile (HR). The centromere positions are represented by blue bars [37]. [file 12711_2021_619_MOESM1_ESM.jpg]

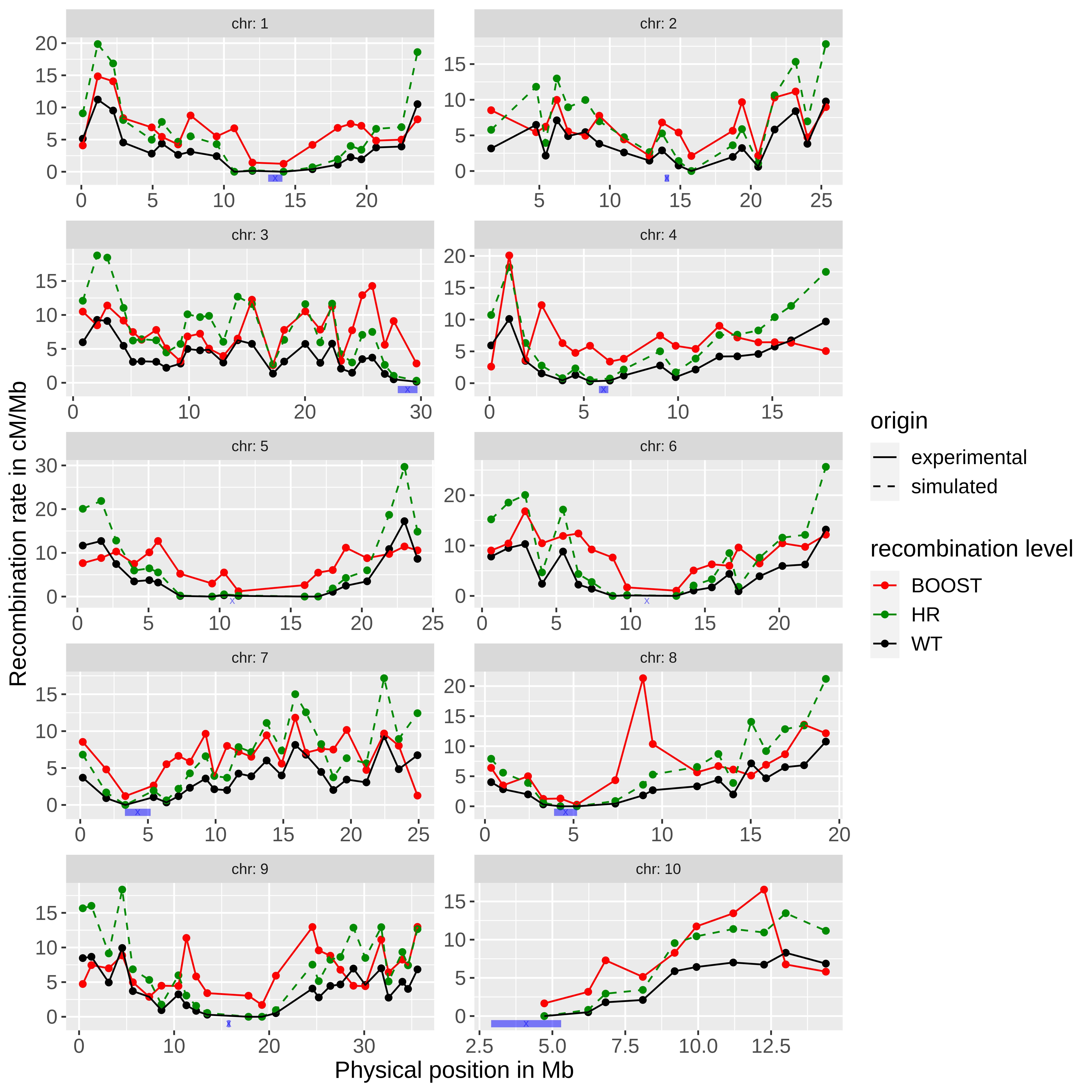

Supplement: Supplementary file 2 — Additional file 2: Figure S2. Male recombination landscapes for the 10 chromosomes of Brassica rapa. The WT, HR and Boost recombination landscapes are represented in black, green and red, respectively. The solid lines represent the profiles obtained from experimental data (WT and Boost, data from [18]) and the dotted line is the simulated profile (HR). The centromere positions are represented using blue bars [37]. [file 12711_2021_619_MOESM2_ESM.jpg]

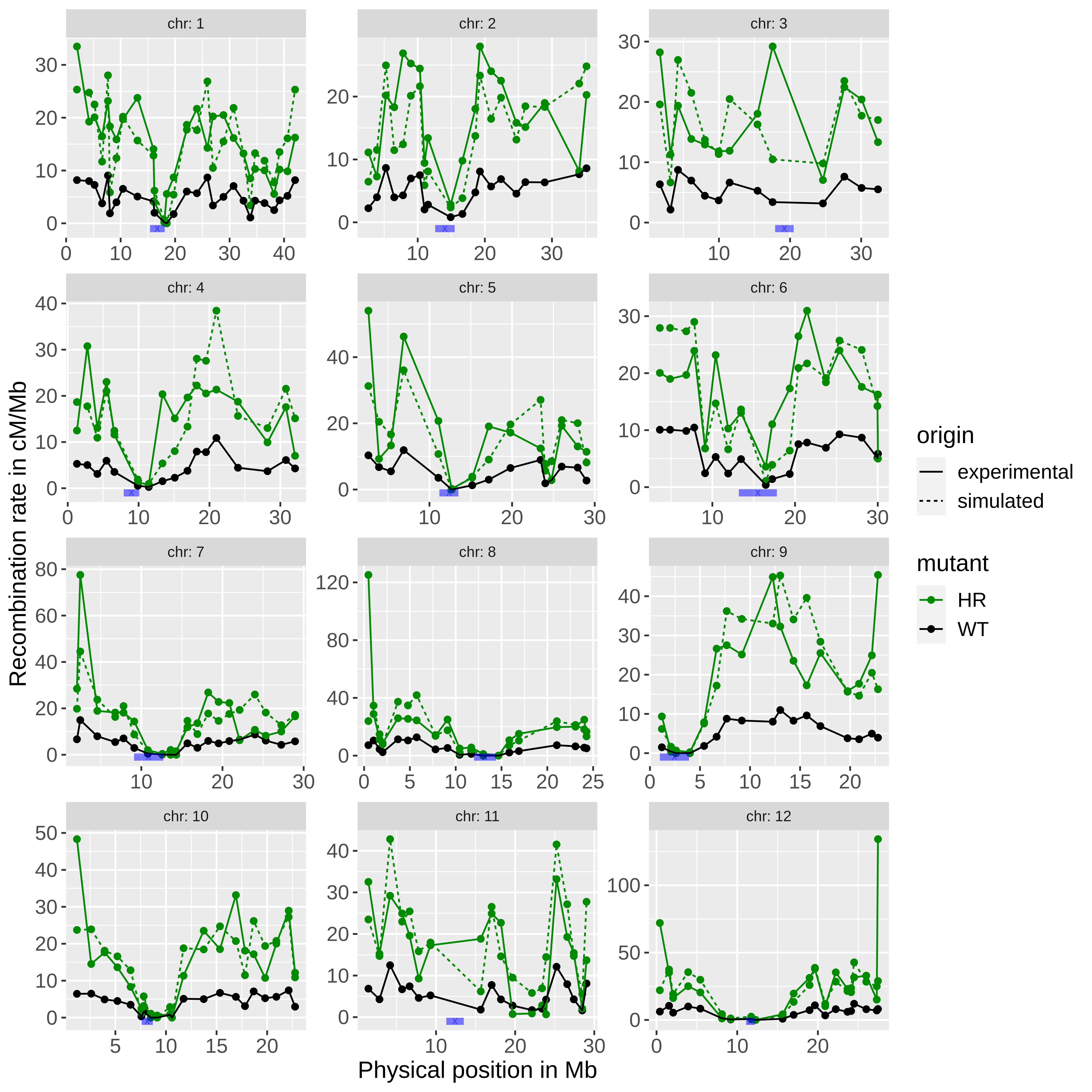

Supplement: Supplementary file 3 — Additional file 3: Figure S3. Recombination landscapes for the 12 chromosomes of Oryza sativa. The WT and HR recombination landscapes are represented in black and green, respectively. Solid lines represent the landscapes obtained experimentally [17] and the dotted line the simulated. The centromere positions are represented by blue bars [38]. This figure shows the effect of HR recombination on the shape of the recombination landscape, as well as the relevance of a homothetic rescaling to approximate HR, thereby justifying this approach for simulating HR in B. rapa. [file 12711_2021_619_MOESM3_ESM.jpg]

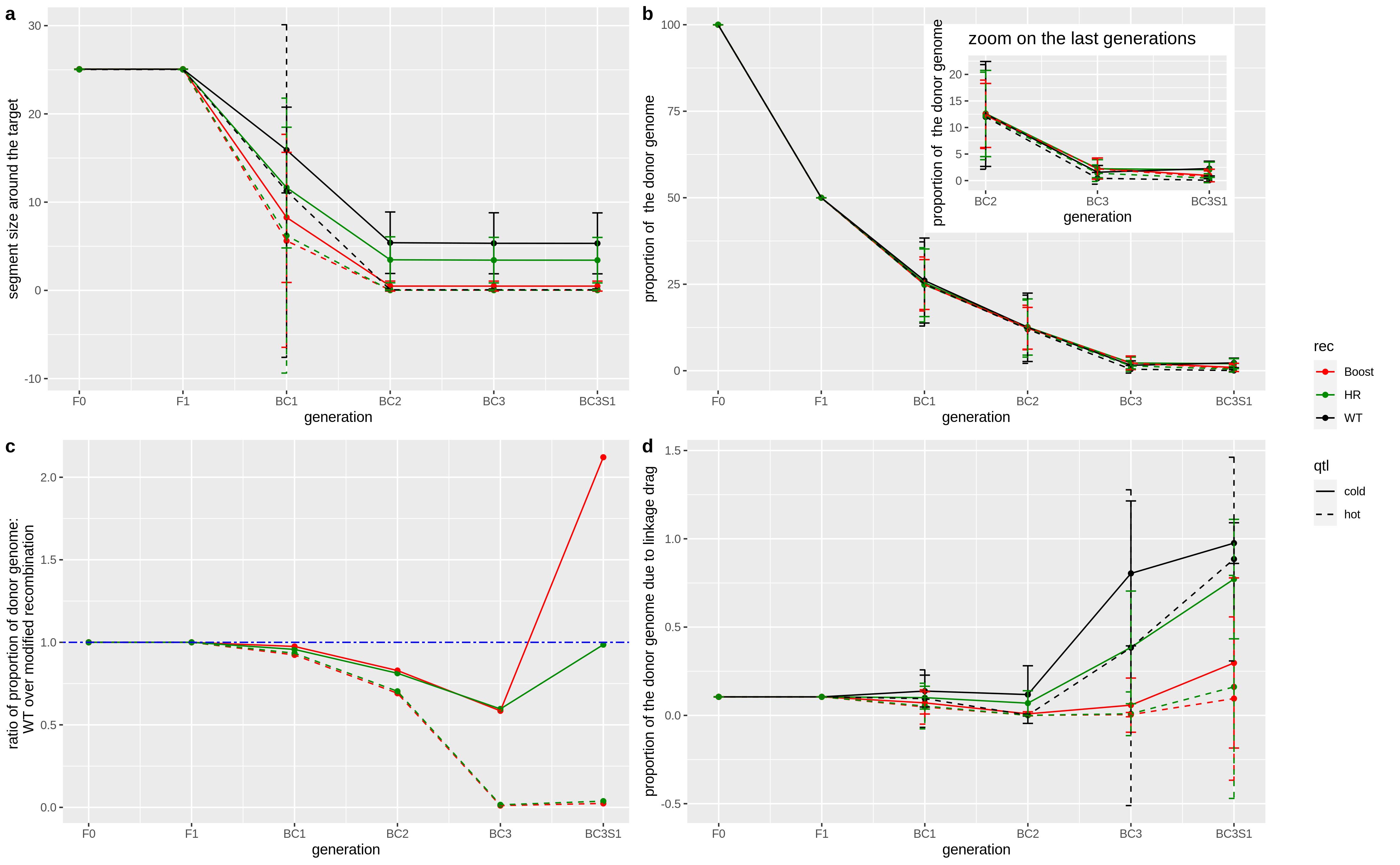

Supplement: Supplementary file 4 — Additional file 4: Table S1 Recombination parameters for the B. rapa male recombination map. Description: Parameters are: the genetic lengths, in centiMorgan, the proportion p of interfering crossovers and the shape parameter, nu, of the gamma distribution used to draw the crossovers under interference. These parameters are defined for a normal recombination rate (wild type, WT) and for increased recombination, either under Boost or HR. The values of p and nu are from [18] for WT and Boost whereas for HR, we assumed no interference (p = 0, nu = 1). The genetic lengths under HR were set to the values measured under Boost. [file 12711_2021_619_MOESM4_ESM.jpg]

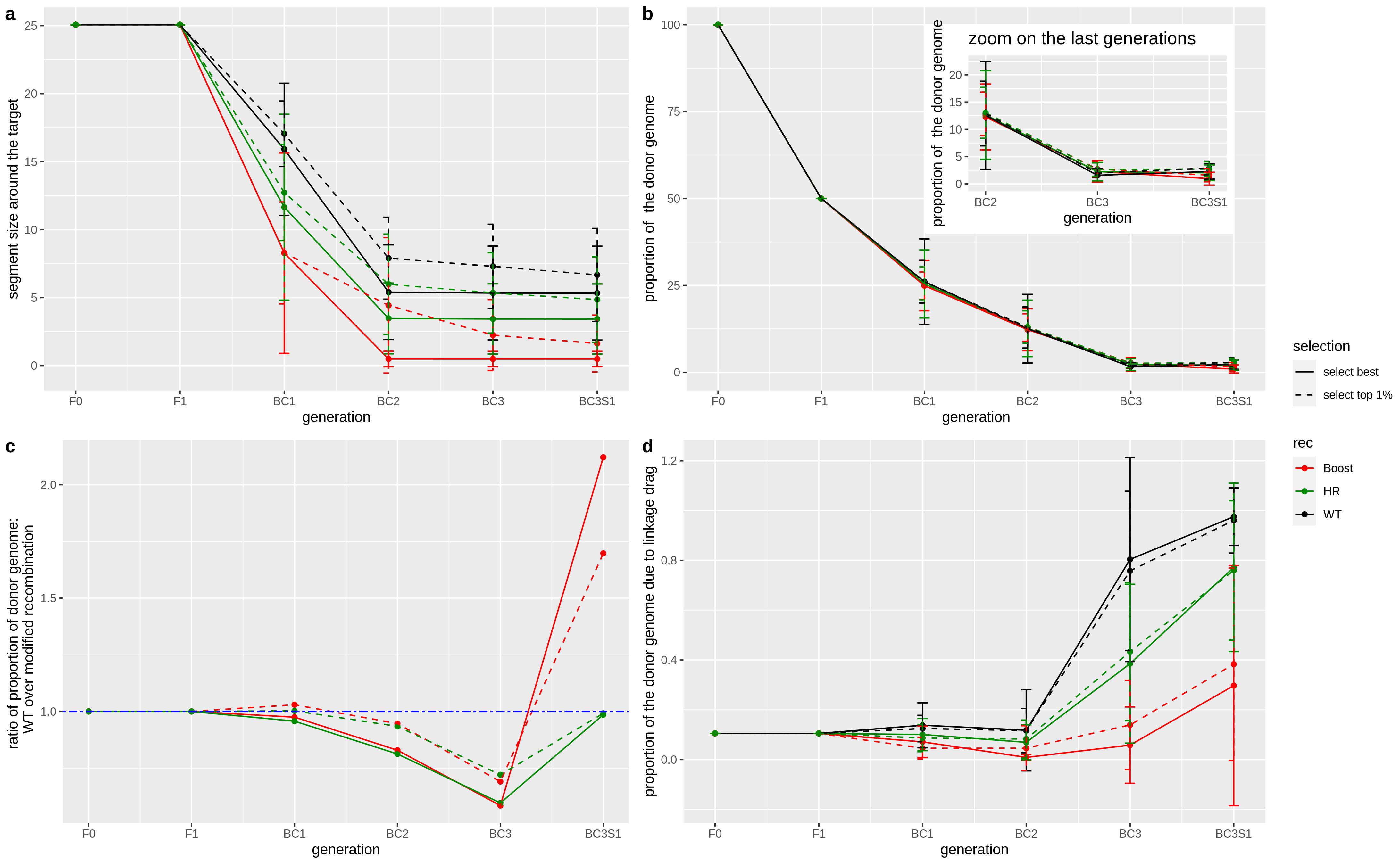

Supplement: Supplementary file 5 — Additional file 5: Figure S4. Effect of the population size. (a) Mean size of the heterozygous segment around the target locus, in Mb, according to generations, in B.rapa. (b) Mean proportion of the donor genome, in percentage, according to generations. The insert represents a zoom on the last generations, from BC2 onwards. (c) Ratio of the mean proportion of donor genome under WT over the proportion under Boost or HR according to generations. A value above 1 means that there is more remaining donor genome in the WT than under modified recombination rates (Boost or HR). (d) Mean proportion of the remaining donor genome that results from the linkage drag, calculated as the part of the remaining donor genome that comes from the heterozygous segment around the target locus, according to generations. The measures for WT, Boost and HR are represented in black red and green, respectively. Different population sizes are shown: 200 plants per generation as dashed lines, 400 as solid lines, and 1000 as dotted lines. In the situations represented in this figure, the target locus is in a cold region, and the selection scheme goes up to BC3S1. The best individual is kept at each generation, following the selection criterion appropriate for each generation (BC1 and BC2: foreground selection, and thereafter background selection). The error bars represent the confidence intervals at 95%. [file 12711_2021_619_MOESM5_ESM.jpg]

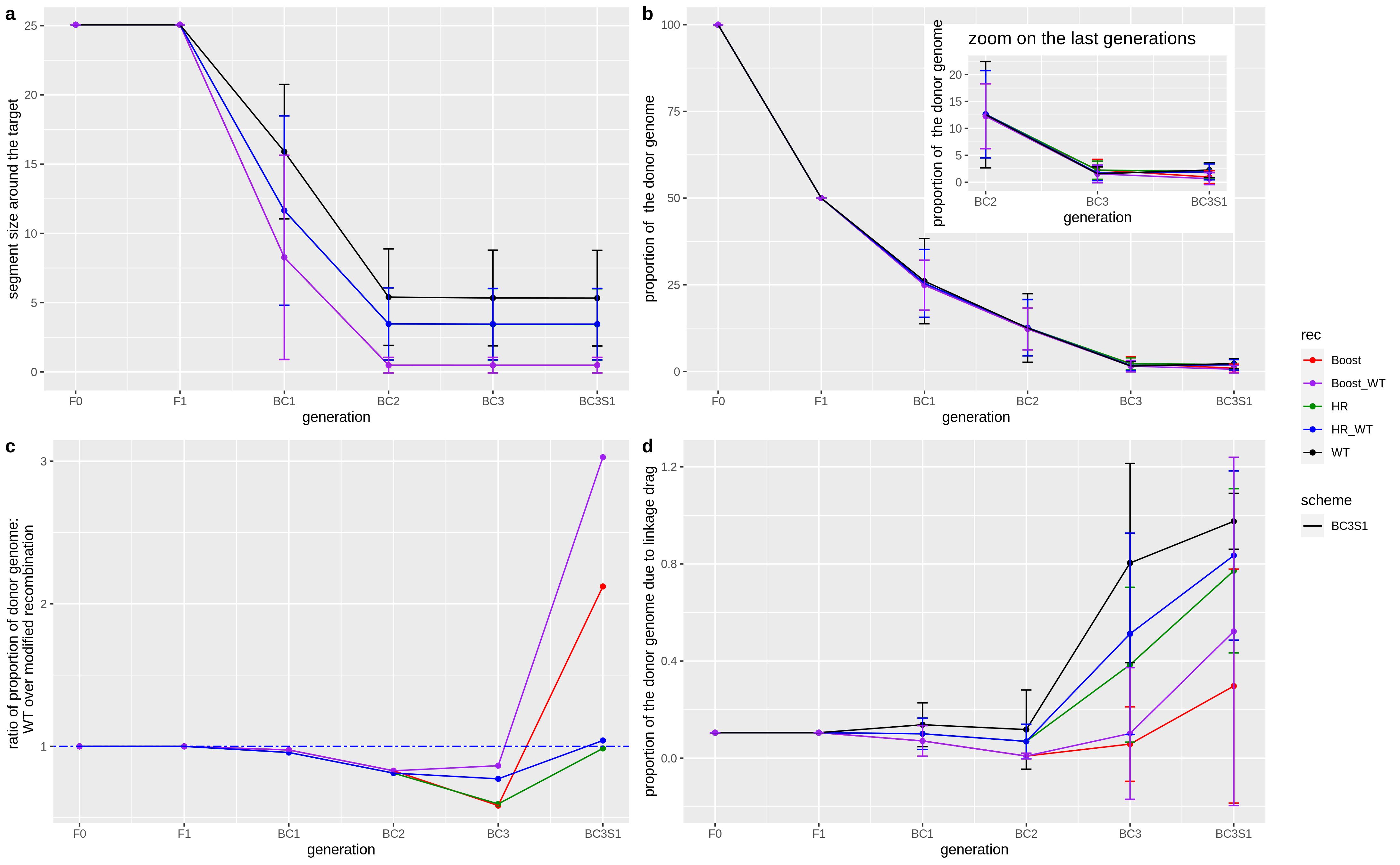

Supplement: Supplementary file 6 — Additional file 6: Figure S5. Effect of the number of generations of backcross used. (a) Mean size of the heterozygous segment around the target locus, in Mb, according to generations, in B.rapa. (b) Mean proportion of the donor genome, in percentage, according to generations. The insert represents a zoom on the last generations, from BC2 onwards. (c) Ratio of the mean proportion of donor genome in the WT to the proportion under Boost or HR according to generations. A value above 1 means that there is more remaining donor genome in the WT than under modified recombination rates (Boost or HR). (d) Mean proportion of the remaining donor genome that results from the linkage drag, calculated as the part of the remaining donor genome that comes from the heterozygous segment around the target locus, according to generations. The measures for WT, Boost and HR are represented in black, red and green, respectively. The selection schemes go up to different numbers of generations: BC3S1 in solid lines, BC4S1 in dashed lines and BC9S1 in dotted lines. In the situations represented in this figure, the target locus is in a cold region and there are 400 plants per generation. The best individual is kept at each generation, following the selection criterion appropriate for each generation (BC1 and BC2: foreground selection, and thereafter background selection). The error bars represent the confidence intervals at 95%. [file 12711_2021_619_MOESM6_ESM.jpg]

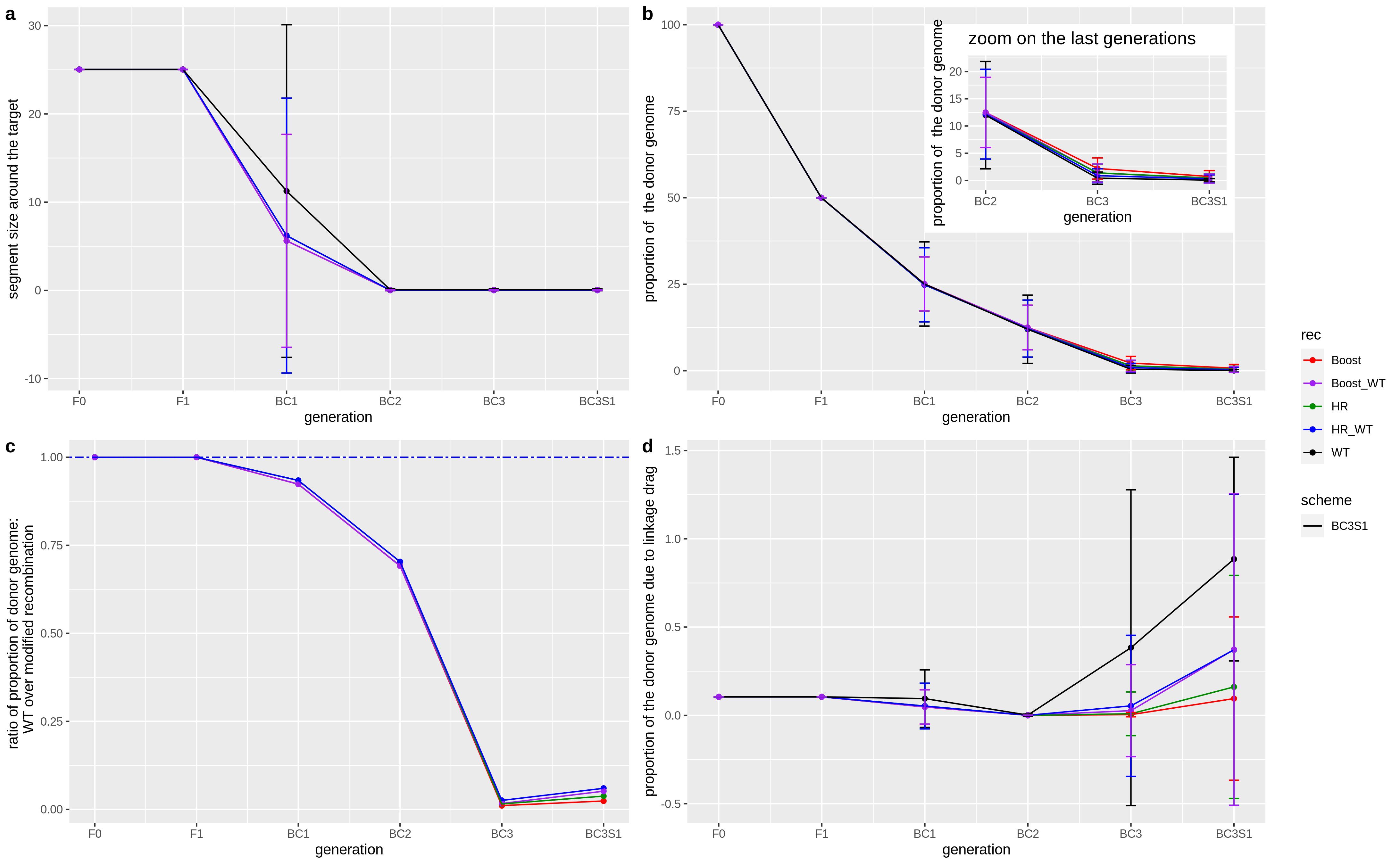

Supplement: Supplementary file 7 — Additional file 7: Figure S6. Introgression in a hot vs cold region. (a) Mean size of the heterozygous segment around the target locus, in Mb, according to generations, in B. rapa. (b) Mean proportion of the donor genome, in percentage, according to generations. The insert represents a zoom on the last generations, from BC2 onwards. (c) Ratio of the mean proportion of donor genome in the WT to the proportion under Boost or HR, according to generations. A value above 1 means that there is more remaining donor genome in the WT than under modified recombination rates (Boost or HR). (d) Mean proportion of the remaining donor genome that results from the linkage drag, calculated as the part of the remaining donor genome that comes from the heterozygous segment around the target locus, according to generations. The measures for WT, Boost and HR are represented in black, red, and green, respectively. The target locus is either in a cold region (solid lines) or in a hot region (dashed lines). HR was not considered when the QTL was in a cold region since the effect of an increased recombination rate mainly influenced the linkage drag and the result under HR was not very different from that in the WT in the cold region. In the situations represented in this figure, there are 400 plants per generation and the selection scheme goes up to BC3S1. The best individual is kept at each generation, following the selection criterion appropriate for each generation (BC1 and BC2: foreground selection, and thereafter background selection). The error bars represent the confidence intervals at 95%. [file 12711_2021_619_MOESM7_ESM.jpg]

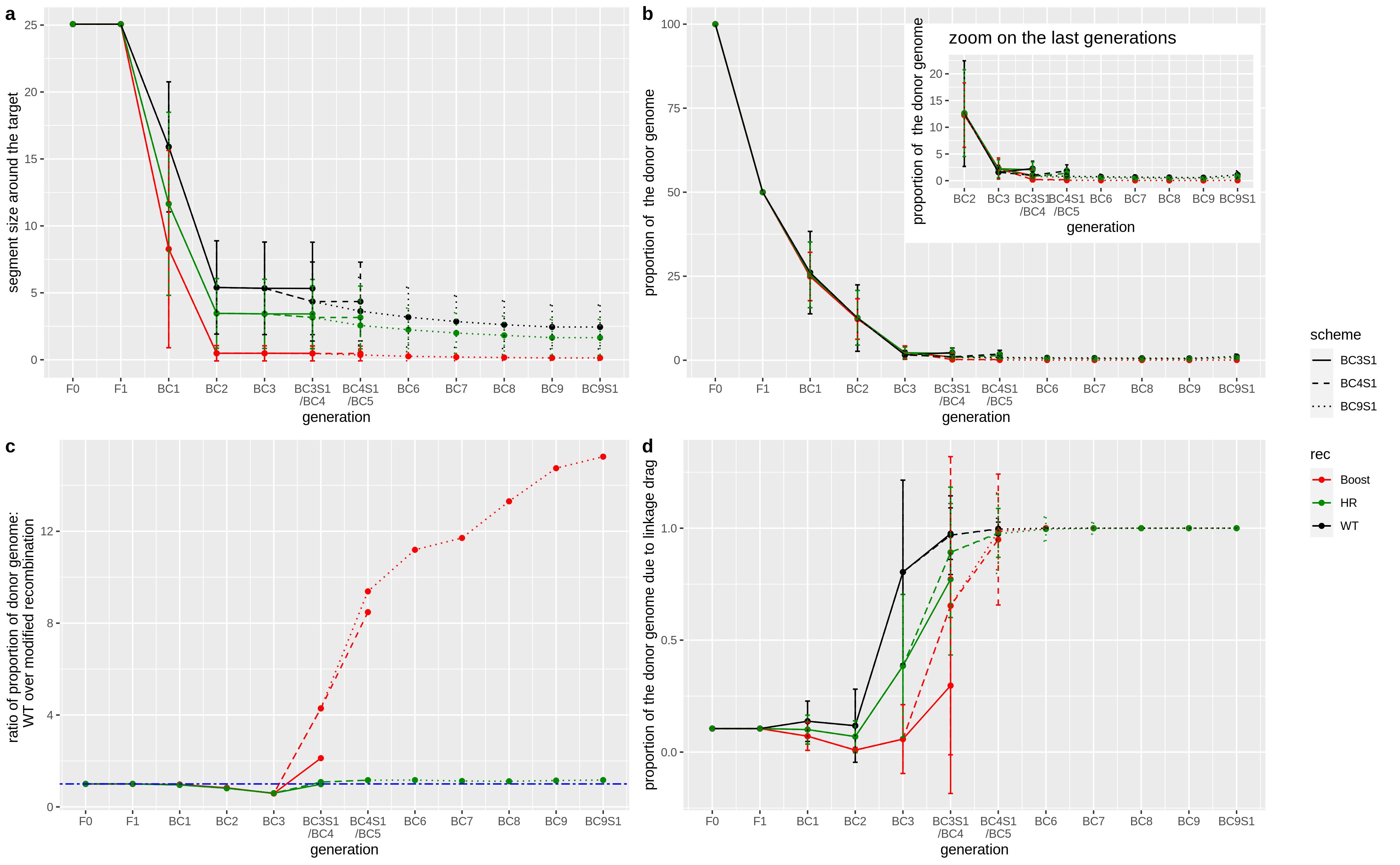

Supplement: Supplementary file 9 — Additional file 9: Figure S8. Effect of switching recombination landscapes when the target locus is in a cold region (a) Mean size of the heterozygous segment around the target locus, in Mb, according to generations, in B.rapa. (b) Mean proportion of the donor genome, in percentage, according to generations. The insert represents a zoom on the last generations, from BC2 onwards. (c) Ratio of the mean proportion of donor genome in the WT to the proportion under Boost or HR according to generations. A value above 1 means that there is more remaining donor genome in the WT than under modified recombination rates (Boost or HR). (d) Mean proportion of the remaining donor genome that results from the linkage drag, calculated as the part of the remaining donor genome that comes from the heterozygous segment around the target locus, according to generations. The measures for WT, Boost and HR are represented in black, red, and green, respectively. The situations in which the recombination rate is switched between foreground (increased recombination) and background (normal recombination) selection are represented in purple for the switch Boost to WT, and in blue for the switch HR to WT. In the situations represented in this figure, the target locus is in a cold region, there are 400 plants per generations and the selection scheme goes up to BC3S1. The error bars represent the confidence intervals at 95%. [file 12711_2021_619_MOESM9_ESM.jpg]

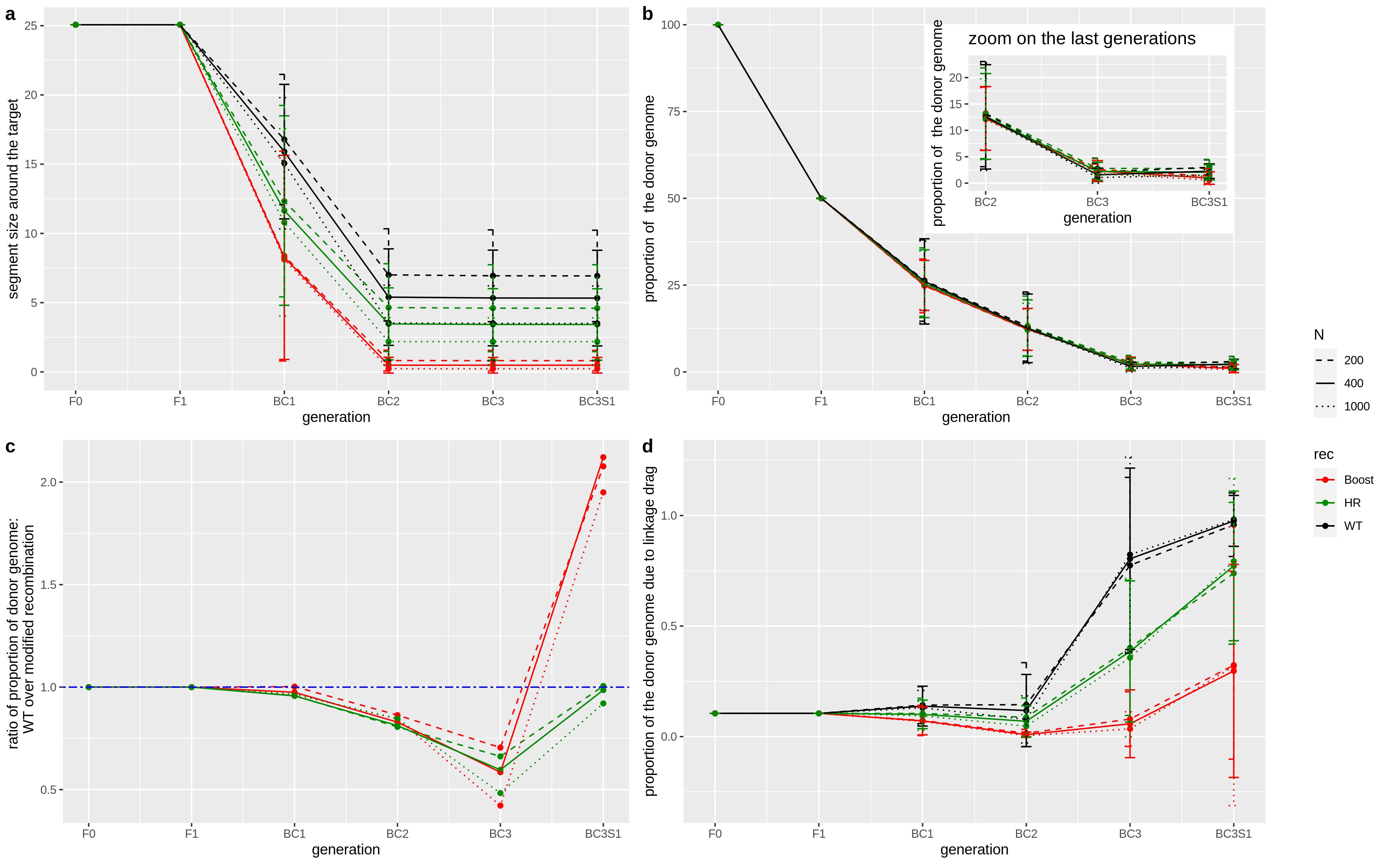

Supplement: Supplementary file 10 — Additional file 10: Figure S9. Effect of switching recombination landscapes when the target locus is in a hot region. (a) Mean size of the heterozygous segment around the target locus, in Mb, according to generations, in B.rapa. (b) Mean proportion of the donor genome, in percentage, according to generations. The insert represents a zoom on the last generations, from BC2 onwards. (c) Ratio of the mean proportion of donor genome in the WT to the proportion under Boost or HR according to generations. A value above 1 means that there is more remaining donor genome in the WT than under modified recombination rates (Boost or HR). (d) Mean proportion of the remaining donor genome that results from the linkage drag, calculated as the part of the remaining donor genome that comes from the heterozygous segment around the target locus, according to generations. The measures for WT, Boost and HR are represented in black, red, and green, respectively. The situations in which the recombination rate is switched between foreground (increased recombination) and background (normal recombination) selection are represented in purple for the switch Boost to WT, and in blue for the switch HR to WT. In the situations represented in this figure, the target locus is in a hot region, there are 400 plants per generations and the selection scheme goes up to BC3S1. The error bars represent the confidence intervals at 95%. [file 12711_2021_619_MOESM10_ESM.jpg]
