# Supplementary material for: Enhancing backcross programs through increased recombination
Source: Genet Sel Evol. 2021 Mar 9;53:25. doi: 10.1186/s12711-021-00619-0 (PMC7941899; doi:10.1186/s12711-021-00619-0)
Supplement: Supplementary file 8 — Additional file 8: Figure S7: Effect of selecting the top 1% best plants rather than the best one. (a) Mean size of the heterozygous segment around the target locus, in Mb, according to generations, in B.rapa. (b) Mean proportion of the donor genome, in percentage, according to generations. The insert represents a zoom on the last generations, from BC2 onwards. (c) Ratio of the mean proportion of donor genome under WT to the proportion under Boost or HR according to generations. A value above 1 means that there is more remaining donor genome in the WT than under modified recombination rates (Boost or HR). (d) Mean proportion of the remaining donor genome that results from linkage drag, calculated as the part of the remaining donor genome that comes from the heterozygous segment around the target locus, according to generations. The measures for WT, Boost and HR are represented in black, red, and green, respectively. Either the best (solid lines) or the best four (top 1%; dashed lines) plants are kept at each generation. In the situations represented in this figure, the target locus is in a cold region, there are 400 plants per generations and the selection scheme goes up to BC3S1. The error bars represent the confidence intervals at 95%. [file 12711_2021_619_MOESM8_ESM.docx]

**Table S1 Recombination parameters for *B. rapa* male recombination map.**

| **Chromosome** | **WT** | | | **Boost** | | | **HR** | | |
| --- | --- | --- | --- | --- | --- | --- | --- | --- | --- |
|  | **genetic length** | **p** | **nu** | **genetic length** | **p** | **nu** | **genetic length** | **p** | **nu** |
| A01 | 87.2 | 1 | 4.891 | 154.3 | 1 | 1.81 | 154.3 | 0 | 1 |
| A02 | 83.9 | 1 | 6.15 | 153.1 | 1 | 2.724 | 153.1 | 0 | 1 |
| A03 | 117.4 | 0.971 | 7.773 | 237.7 | 1 | 2.851 | 237.7 | 0 | 1 |
| A04 | 68.8 | 1 | 4.369 | 124.2 | 1 | 4.296 | 124.2 | 0 | 1 |
| A05 | 98.9 | 0.93 | 13.46 | 170.2 | 0.903 | 4.193 | 170.2 | 0 | 1 |
| A06 | 101.5 | 0.94 | 7.356 | 197.5 | 1 | 3.384 | 197.5 | 0 | 1 |
| A07 | 90.1 | 0.932 | 17.801 | 166.2 | 1 | 3.813 | 166.2 | 0 | 1 |
| A08 | 68 | 0.929 | 23.329 | 133.9 | 1 | 2.591 | 133.9 | 0 | 1 |
| A09 | 123.8 | 0.905 | 6.886 | 228.9 | 0.999 | 2.688 | 228.9 | 0 | 1 |
| A10 | 52.9 | 1 | 11.693 | 86 | 1 | 3.106 | 86 | 0 | 1 |

Recombination parameters used for *B. rapa* male recombination map. The parameters are the genetic lengths, in centiMorgans, the proportion p of interfering crossovers and the shape parameter, nu, of the gamma distribution used to draw the crossovers under interference. These parameters are defined for normal recombination (wild type, WT) and for increased recombination, either via Boost or HR. These values were taken from Pelé *et al.* 2017 for the WT and Boost values and calculated using the fact that we supposed the same global increase of recombination for Boost and HR (same genetic lengths) and that HR results in no interference (p = 0 and nu = 1).
